# Microencapsulation of *Salmonella*-Specific Bacteriophage Felix O1 Using Spray-Drying in a pH-Responsive Formulation and Direct Compression Tableting of Powders into a Solid Oral Dosage Form

**DOI:** 10.3390/ph12010043

**Published:** 2019-03-22

**Authors:** Gurinder K. Vinner, Zahra Rezaie-Yazdi, Miika Leppanen, Andrew G.F. Stapley, Mark C. Leaper, Danish J. Malik

**Affiliations:** 1 Department of Chemical Engineering, Loughborough University, Loughborough LE11 3TU, UK; G.Vinner@lboro.ac.uk (G.K.V.); Z.Rezaie-Yazdi@lboro.ac.uk (Z.R.-Y.); a.g.f.stapley@lboro.ac.uk (A.G.F.S.); M.C.Leaper@lboro.ac.uk (M.C.L.); 2 Department of Physics, Department of Biological and Environmental Science, Nanoscience Center, University of Jyväskylä, 40014 Jyväskylä, Finland; miika.j.leppanen@jyu.fi

**Keywords:** antibiotic resistance, bacteriophages, direct compression, microparticles, pH-responsive, spray drying, *salmonella*, tablets

## Abstract

The treatment of enteric bacterial infections using oral bacteriophage therapy can be challenging since the harsh acidic stomach environment renders phages inactive during transit through the gastrointestinal tract. Solid oral dosage forms allowing site-specific gastrointestinal delivery of high doses of phages, e.g., using a pH or enzymatic trigger, would be a game changer for the nascent industry trying to demonstrate the efficacy of phages, including engineered phages for gut microbiome modulation in expensive clinical trials. Spray-drying is a scalable, low-cost process for producing pharmaceutical agents in dry powder form. Encapsulation of a model *Salmonella*-specific phage (*Myoviridae* phage Felix O1) was carried out using the process of spray-drying, employing a commercially available Eudragit S100^®^ pH-responsive anionic copolymer composed of methyl methacrylate-*co*-methacrylic acid formulated with trehalose. Formulation and processing conditions were optimised to improve the survival of phages during spray-drying, and their subsequent protection upon exposure to simulated gastric acidity was demonstrated. Addition of trehalose to the formulation was shown to protect phages from elevated temperatures and desiccation encountered during spray-drying. Direct compression of spray-dried encapsulated phages into tablets was shown to significantly improve phage protection upon exposure to simulated gastric fluid. The results reported here demonstrate the significant potential of spray-dried pH-responsive formulations for oral delivery of bacteriophages targeting gastrointestinal applications.

## 1. Introduction

The emergence of antibiotic resistance in pathogenic bacteria is a serious global health threat. Common enteric bacterial pathogens are becoming progressively resistant to frontline antibiotics. The pipeline for the development of new classes of broad-spectrum antibiotics is not looking promising [1]. In addition to treating gastrointestinal infections in humans, a safe and low-cost strategy to reduce pathogen carriage in livestock and poultry is also needed. National health agencies are increasingly banning general antibiotic use in animals grown for human consumption, e.g., see the European Union (EU) directive on additives for use in animal nutrition [2]. There is an increasing awareness of the need to move away from broad-spectrum antibiotics and use more specific treatments which do not cause dysbiosis of the microbiome [3].

Lytic bacteriophages (phages) are viruses that infect and kill bacteria including antibiotic-resistant ones in a highly species-specific manner. Commonly occurring gastrointestinal infections are caused by several types of bacteria including *Clostridium difficile*, *Escherichia coli*, *Salmonella* spp., and *Vibrio cholera* [4]. It is estimated that *Salmonella* alone accounts for 1.2 million foodborne illnesses in the United States, with 23,000 hospitalisations and 450 deaths costing an estimated 365 million dollars in medical costs each year [5]. Increasing centralisation and industrialisation of food supply increases the risk of distribution of these hardy organisms. Antimicrobial resistance to “first-line” drugs is increasingly common among *Salmonella* worldwide [6,7,8,9]. In animals, decolonisation of the gastrointestinal tract from *Salmonella* may be beneficial for biocontrol to reduce dissemination of harmful bacteria through the food chain, e.g., lairage-associated *Salmonella* transmission in pigs [10]. Phages incorporated in solid oral dosage forms may be mixed in with animal feed for prophylactic or therapeutic applications.

The use of phage therapy is a particularly promising alternative to using broad-spectrum antibiotics for acute enteric infections, because typically in such cases intestinal concentration of the infecting bacteria is high and the causative agent and strain may be suitably diagnosed using rapid diagnostic tools. Solid oral dosage forms that are capable of reliably delivering a therapeutically high phage dose to the site of infection is a major barrier for the treatment of gastrointestinal infections [11,12,13,14]. Phages are biological entities requiring protection from stresses typically encountered during manufacturing and storage [15]. The harsh conditions encountered in the human stomach (pH ~1), as well as exposure to bile and digestive enzymes in the gastrointestinal tract, render phages inactive [12,13,16,17,18,19,20]. Previous studies on phage encapsulation focused on extrusion methods, e.g., to produce hydrogel microparticles with variable levels of acid protection [16,17,21]. Recently, more sophisticated microfluidic methods were used, giving precise control over the fabrication of microcapsules. Such methods, however, are difficult to scale-up, costly, and better suited for high-value products [12,13]. There is a need for scalable low-cost methods for producing stable oral dosage forms for delivering bacteriophages to the gastrointestinal tract.

Spray-drying is an industrially acceptable process used to manufacture dry powder forms carrying bioactive agents such as proteins, peptides, attenuated antibodies, and phages [22]. Spray-drying was previously used for producing phage-containing powders in sugar formulations suitable for pulmonary delivery [23,24,25]. Published studies employing spray-drying to produce phage-containing powders with pH-responsive characteristics are relatively rare; however, a previous study employing spray-dried *E. coli* phages using pure Eudragit S100^®^ showed a ~1 log loss in phage titre in the final spray-dried powder [26]. The *E. coli* phage titre was shown to fall significantly (between 2–3 log reduction) during storage over a period of one year at 20 °C for different phages evaluated in the study [26]. However, the focus of previous research was not on optimising the spray-drying conditions, and evaluation of the effects of excipients on phage viability and storage stability were not adequately addressed. Research is, therefore, needed to evaluate suitable formulations and optimum spray-drying conditions to produce acid stable solid dosage forms for enteric delivery, and these are addressed in this study. Dry powder forms are favoured due to their ease of handling and long-term storage stability, e.g., at ambient temperatures, thereby avoiding the need for a cold supply chain for storage. Encapsulating phages in a stable dry powder form opens possibilities for their use in oral solid dosage forms, e.g., using direct compression to produce tablets for enteric delivery [27].

Commercial spray-dryers typically operate at high temperatures with powders exposed to temperatures around or exceeding 100 °C [28]. This ensures low residual moisture content in the dried product which impacts positively on powder handling and storage stability. Exposing phages to elevated temperatures can be detrimental to phage viability, resulting in the loss of phage titre in the final dried powder. Phages were previously spray-dried at low outlet temperatures using small laboratory-scale spray-dryers with outlet temperatures typically between 40 and 60 °C and using excipients such as trehalose to provide protection from thermal stresses [23,25]. Good excipients such as trehalose prevent or minimise the effect of thermal stress on the active agents and act as water-replacing agents. Phage proteins may undergo irreversible damage due to the removal of water, which is essential for the maintenance of the hydrogen bonds necessary to stabilise their secondary structure [24]. Trehalose replaces these hydrogen bonds and vitrifies the phages, yielding dry powders with high glass transition temperatures [29].

The aim of the present study was to investigate the effect of spray-drying temperatures and formulation parameters, varying the amount of trehalose and the pH-responsive polymer Eudragit S100^®^ to produce stable dry powders having a high amount of encapsulated phage and good storage stability. The spray-dried powders were tableted using a direct compression process. Powders and tablets containing phages were exposed to simulated gastric fluid to evaluate acid protection. The work reported here allows evaluation of the suitability of these low-cost scalable methods for phage encapsulation in oral solid dosage forms. This study addresses the urgent unmet need for the development of scalable delivery methods to facilitate translation of phages from bench-to-bedside in order to demonstrate the effectiveness of phage therapy in both humans and animals.

## 2. Materials and Methods

### 2.1. Model Bacterium and Phage

*Salmonella* and phage Felix O1 were used as the model bacterium and phage for this study. *Salmonella enterica* ATCC19585 was purchased from LGC standards, EU. Phage Felix O1 was kindly donated by Dr Cath Rees, University of Nottingham, UK [30]. An *S. enterica* strain was used to propagate and enumerate Felix O1.

Bacterial growth and phage propagation were carried out using previously published methods [13]. Briefly, a log-phase culture of *Salmonella* at an optical density (OD) of 0.2 (this typically equates to a viable cell count of ~10^8^ colony-forming units (CFU)/mL) was inoculated with Felix O1 at a multiplicity of infection (MOI) of 0.01. The lysate was centrifuged at 2000× *g* and filtered using a 0.2-µm pore size in-line syringe filter (Millipore, Watford, UK) and stored at 4 °C until further use. Plaque assays were used to enumerate phage concentration employing the double overlay agar method; serial dilutions of phages were spotted on a bacterial lawn overlay. All measurements were performed in triplicate. The plaque-forming units (PFU) were counted after incubation for 24 h at 37° C.

### 2.2. Spray-Drying Conditions and Formulations

Eudragit S100 was kindly supplied by Evonik Germany. d-(+)-Trehalose dihydrate was purchased from Fisher Scientific (Loughborough, UK). Solutions containing different excipient (Eudragit S100 or trehalose) amounts were dissolved in 500 mL of deionised distilled water (dH_2_O). For ease of presentation, the following nomenclature is used in the manuscript: the amount of polymer (Eudragit, denoted as “P”) to sugar (trehalose, denoted as “S”) corresponds to the dissolved percentage (*w*/*v*) of polymer and sugar in the solutions used for spray-drying, e.g., PS21 refers to 2% (*w*/*v*) polymer and 1% (*w*/*v*) sugar in the solution. The following formulations with different proportions of trehalose and polymer were evaluated in the study: PS04 (4% *w*/*v* trehalose), PS30 (3% *w*/*v* polymer), PS32 (3% *w*/*v* polymer, 2% *w*/*v* trehalose), PS21 (2% *w*/*v* polymer, 1% *w*/*v* trehalose), and PS24 (2% *w*/*v* polymer, 4% *w*/*v* trehalose).

In order to dissolve Eudragit, the pH of the water was changed to alkaline (pH 12) via addition of 4 M NaOH (Fisher Scientific, UK) to allow polymer dissolution, followed by pH adjustment to pH 7 using 0.1 M HCl prior to addition of trehalose powder, its dissolution, and then addition of bacteriophages to the solution. For each formulation, typically 1% (*v*/*v*) high-titre phage Felix O1 (~10^11^ PFU/mL) was added to the solution, yielding phage titres of ~10^9^ PFU/mL in the final formulations. The phage-containing solutions were spray-dried using a commercially available Labplant spray-dryer SD-06 (Labplant, UK Limited), which is a co-current dryer with a pneumatic atomiser and a cylindrical drying chamber of dimensions 215 mm outer diameter and 420 mm height. The air exit stream was passed through a high-efficiency particulate air (HEPA) filter prior to discharge. The diameter of the atomization nozzle used throughout the work was 0.5 mm with the measured feed liquid flow rate at 280 mL∙h^−1^ and a drying gas air flow rate of ~20 L∙s^−1^. The air inlet temperatures were set at 100 °C, 120 °C, 150 °C, and 180 °C resulting in corresponding air outlet temperatures of 56 ± 2 °C, 66 ± 2 °C, 82 ± 2 °C, and 96 ± 2 °C, respectively. The outlet temperature is only indicative of the highest temperature the phages could be exposed to as dry powders in the collection bottle; temperature in the collection bottle varied between 40 and 60 °C.

### 2.3. Powder Storage

Following collection, the phage-containing spray-dried powders were stored in sealed screw top bottles. These were stored at either 4 °C or 23 °C for a period of up to three months. At specific time intervals, 0.1 g of powder was removed and dissolved in simulated intestinal fluid (SIF) (10 mg/mL of pancreatin in 0.5 mM KH_2_PO_4_, pH 7) and the phage titre was enumerated using a plaque assay (described above). Similarly, experiments involving powder or tablet exposure to simulated gastric fluid (SGF) (composition 3.2 mg/mL of pepsin in 0.2 M NaCl, pH adjusted to pH 2 using 5 M HCl) involved weighing a known amount of powder or tablet (typically 0.3 g) and these were subsequently exposed to 10 mL of SGF for 2 h at 37 °C. Following SGF exposure, the powder or tablet samples were centrifuged at 2000× *g* to pellet the suspended solids. Supernatant SGF was removed using a pipette. Then, 10 mL of SIF was added to the pelleted solid material, which was gently vortexed to resuspend the solids, before being left in an orbital incubator shaker (Certomat, Sartorius, UK) at 37 °C and 120 rpm. Complete dissolution of the powders (SIF exposure for 3 h at 37 °C) and tablets (SIF exposure for 5 h at 37 °C) was achieved. Thereafter, 10 µL of the supernatant was removed using a micropipette; then, the sample was serially diluted and plaque assays were performed to measure phage release.

### 2.4. Direct Compression Tableting of Spray-Dried Powders

Spray-dried powders at an inlet temperature of 150 °C were used for tableting using a Riva Minipress MII (UK) tabletting machine. Approximately 0.3 g of powder was loaded into the punch hole and compressed at a force of 5 kN. The produced tablets were weighed before being stored at 4 °C in sealed tubes for further analysis.

### 2.5. Ion Microscopy

To analyse the morphology of spray-dried powders, a representative sample from formulation PS21 spray-dried at an inlet temperature 150 °C was examined with ion microscopy. Dried powder was applied on carbon tape attached to a sample stub, and any excess was blown away. A Zeiss Orion NanoFab (University of Jyväskylä) with Ne^+^ beam and acceleration voltage of 10 kV was used to cut the microparticle in half. Milling was done using a 45° tilted angle by setting the reduced raster scan rectangle over the area to be removed and scanned until the material disappeared. Ion current values used were typically ~20 pA, resulting in a total processing time of about 1 h. Following sample cutting by milling, the sample stage was rotated through 180° and the cross-section was imaged with He^+^. An acceleration voltage of 33 kV, a current of 0.20 pA, 32 line averages, and 1 µs of dwell time were used for He^+^ imaging. Flood-gun charge compensation was used during both milling and imaging.

### 2.6. Moisture Content

The average moisture content (wet basis) of the spray-dried powder was measured gravimetrically. A known mass of sample (approximately 0.5 g) was placed in a dry ceramic crucible and dried in a vacuum oven at 120 °C for a period of 24 h. Measurements were done in triplicate. The sample was then removed, cooled over silica gel in a sealed desiccator, and immediately weighed to limit water absorption from the atmosphere. The initial and final masses were then used to calculate the wet basis moisture content.

### 2.7. Particle Size Distribution

A Coulter LS 130 Particle Size Analyzer (Beckman Coulter Inc., High Wycombe, UK) was used to determine the particle size distribution of the spray-dried powders. The device is laser-modulated and uses the optical model Fraunhofer to detect laser diffraction caused by particles. A representative sample from formulation PS21 was taken, loaded into a glass sample containing *n*-hexane (Sigma Aldrich, Gillingham, U.K.) as a suspending medium. The cell was equipped with a stirrer to keep the particles suspended in the solvent. The volume size and cumulative distribution were measured.

### 2.8. Differential Scanning Calorimetry (DSC) Analysis of Spray-Dried Trehalose Powders

Typically, ~15 mg of the spray-dried powder PS04 (stored upon collection in a dry atmosphere over silica gel in a sealed desiccator) was placed in a pre-weighed DSC aluminium pan. The pan was then hermetically sealed and weighed to 0.1-mg accuracy. The sample was placed in a Q10 DSC (TA Instruments, Crawley, Sussex, UK) and scanned from 25 to 120 °C at a programmed heating rate of 10 °C/min. An empty pan was used as a reference. All DSC measurements were carried out in duplicate. The DSC instrument was calibrated for enthalpy and temperature using an indium standard at the same scan rate. The glass transition temperature was computed from each thermal curve.

### 2.9. Statistical Analysis

Statistical analysis was carried out using Minitab version 18 (USA). Two-sample *t*-tests were performed (*n* = 3) with reporting of *p* < 0.05 as statistically significant. Where multiple tests were done, the value of alpha was adjusted using the Bonferroni correction. Error bars represent a single standard deviation for the mean values of the replicates.

## 3. Results

### 3.1. Optimisation of Spray-Drying Formulation of Felix O1 Phage

**Figure 1:**
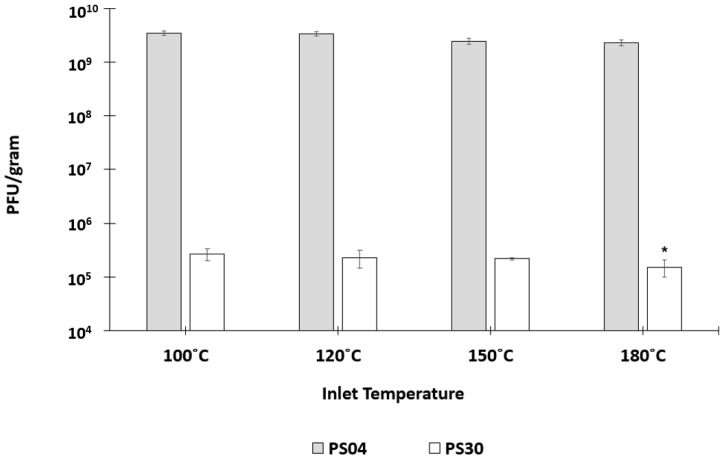
Phage Felix O1 spray-dried at varying inlet air drying temperatures in formulations PS04 and PS30 (see text for description) followed by complete release of phages in simulated intestinal fluid (SIF) (titre measured after 3 h of exposure to SIF). The phage titre of feed solution used for spray-drying was 5 × 10^9^ PFU/mL, equating to a theoretical final phage concentration of ~1.7 × 10^9^ PFU/g. * Indicates significant difference in means for samples at a given temperature compared with the spray-dried sample at 100 °C for the same formulation (*p* < 0.05) using a two-sample *t*-test. Error bars represent one standard deviation; all measurements were done in triplicate (*n* = 3).

Felix O1 at a phage titre of 5 × 10^9^ PFU/mL was spray-dried individually in trehalose (PS04) and Eudragit alone (PS30). The moisture content of PS04 spray-dried powders was typically in the range of ~2–10% (*w*/*w*) and less than 5% (*w*/*w*) for inlet drying temperatures 150 °C and above (Figure S1, Supplementary Materials). The moisture content of PS30 spray-dried powders was considerably higher in the range of ~15–25% (*w*/*w*). The effect of inlet air temperature on phage survival following the spray-drying process was evaluated. The spray-dried phage-containing powders were exposed to SIF for a period of 3 h or until complete dissolution of powder, and the resulting phage concentration was measured (Figure 1). Trehalose was found to be an excellent excipient for the protection of Felix O1 phages exposed to outlet temperatures which were varied between 56 and 96 °C. Phage titre of the feed solution used for spray-drying was 5 × 10^9^ PFU/mL, equating to a theoretical phage concentration of ~1.7 × 10^9^ PFU/g using the assumption that all the phage virions present in the original solution remained viable in the final collected powder, accounting for the amount of dissolved solids in the feed solution. There was no measurable loss in phage titre in the PS04 powders for the entire range of drying temperatures evaluated (*p* > 0.05), and the mean phage titre in the powders was the same as the theoretical yield of ~1 × 10^9^ PFU/g. Felix O1 phages spray-dried in pure Eudragit S100 (PS30) resulted in a significant loss in phage titre; typically, a ~4 log reduction in phage titres was observed at all temperatures post spray-drying in PS30. A two-sample *t*-test of means indicated that the phage titre in the powders at 180 °C was lower in comparison with the phage yield in PS30 powders spray-dried at lower temperatures. For example, at 100 °C, the 95% confidence interval for the mean was between 1.8 × 10^5^ and 3.6 × 10^5^ PFU/g, compared with between 4.6 × 10^4^ and 2.6 × 10^5^ PFU/g at 180 °C (*p* < 0.05). There was no difference in phage titres for PS30 powders spray-dried at 120 °C and 150 °C compared with 100 °C (*p* > 0.05).

**Figure 2:**
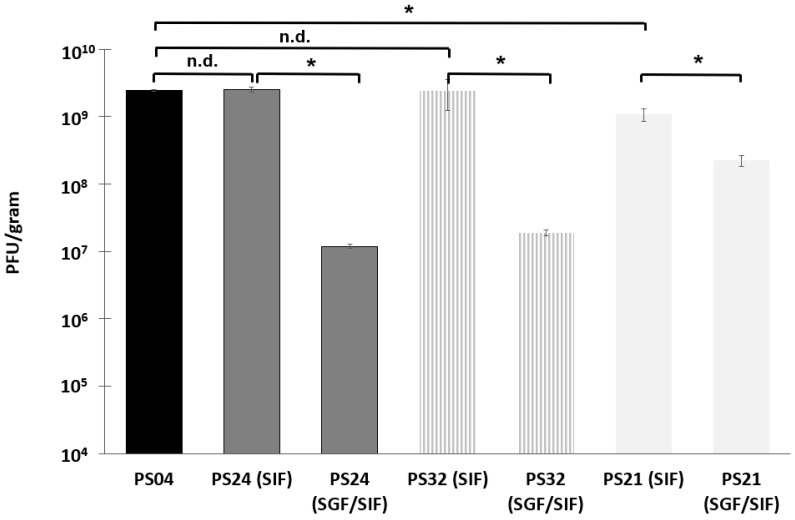
Concentration of encapsulated phage Felix O1 released from spray-dried powders after complete dissolution in SIF (~3 h). Samples labelled SIF were exposed to SIF only without acid exposure, whereas samples labelled simulated gastric fluid (SGF)/SIF were first exposed to simulated gastric fluid (pH 2) for 2 h, subsequently centrifuged to remove the acid supernatant, and then SIF (pH 7) was added to the sample to dissolve the polymer. All formulations were spray-dried at 150 °C inlet temperature corresponding to 82 °C outlet temperature. * Indicates significant difference in means (*p* < 0.05) using a two-sample *t*-test; n.d. means no difference (*p* > 0.05). Error bars represent one standard deviation; all measurements were done in triplicate (*n* = 3).

The effect of combining trehalose (thermal protection and desiccation resistance) and Eudragit S100^®^ (pH resistance and pH trigger for release) on phage survival following spray-drying at 150 °C was examined using formulations with varying proportions of trehalose (Figure 2). In comparison with phage survival in formulation PS04 (100% trehalose, 2.4 × 10^9^ PFU/g), phage titres remained high at 2.5 × 10^9^ PFU/g (PS24, 67% trehalose), 1.8 × 10^9^ PFU/g (PS32, 40% trehalose), and 1.1 × 10^9^ PFU/g (PS21, 33% trehalose). A two-sample t-test comparing the means for samples PS24 and PS32 with sample PS04 showed no statistical difference in means. However, there was a difference for PS21 (95% confidence interval for difference in means was 5 × 10^8^–2.2 × 10^9^ PFU/g higher for PS04 compared with PS21), i.e., similar in magnitude to the titre of PS21. This suggests that the proportion of trehalose in the polymer formulations does affect phage survival during spray-drying with higher trehalose proportions in the powders PS24 and PS32 yielding higher phage titres.

Spray-dried formulations PS24, PS32, and PS21 were all exposed to SGF for 2 h followed by quantification of the remaining viable phage after dissolution of the polymer in SIF. Felix O1 phages encapsulated in formulations PS24 and PS32 were not sufficiently protected from acid exposure at pH 2. Phage titre in the powders fell by ~2 log from ~10^9^ PFU/g to ~10^7^ PFU/g for PS24 and PS32. The sample with a higher proportion of polymer content (PS21) showed a considerably smaller reduction in phage titre upon acid exposure from 1.1 × 10^9^ PFU/g to 2.2 × 10^8^ PFU/g, i.e., around a 1 log reduction. The higher loss of phage viability in sample PS24 and PS32 compared with PS21 may be attributed to the higher proportion of trehalose (67% trehalose in PS24 and 40% trehalose in PS32 compared with 33% in PS21) dispersed in the microparticle shell material; sugar may readily dissolve upon exposure to SGF, exposing phages to the acid environment.

### 3.2. Powder Characterisation

**Figure 3:**
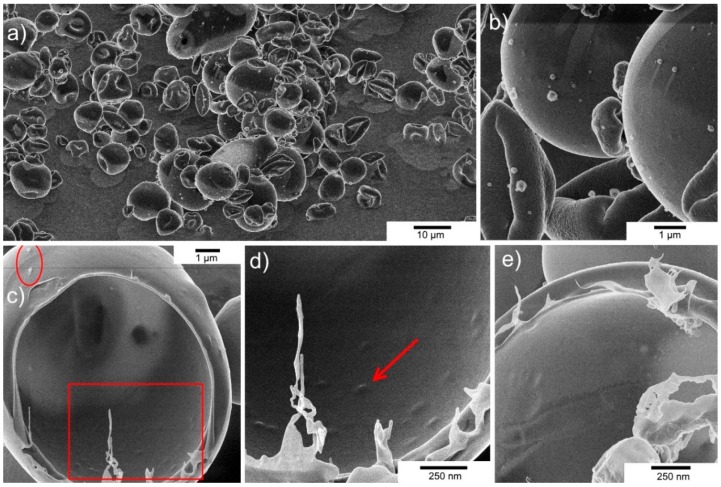
Helium ion microscopy (HIM) images of spray-dried PS21 microparticles (inlet drying temperature 150 °C). (**a**) Spray-dried microparticles were typically <10 µm in size and did not display surface defects such as blow holes. (**b**) Some particles were spherical, whereas others were flattened spheres, and some had a lens-shaped appearance. (**c**) A spherical particle about 10 µm in size was cut in half using a neon ion beam and, after 180° rotation, was imaged with a helium ion beam. Two phage virion particles can be seen in the top left-hand corner (red circle). (**d**) Expanded view of red box area shown in frame (**c**). Bumps in the inside wall of the sphere were found in microparticles containing phages imaged using a higher magnification (red arrow). (**e**) Inside wall of a control microparticle not containing phages.

Helium ion microscopy (HIM) imaging of the spray-dried powders (PS21) revealed the morphology of the microparticles (Figure 3). Spray-dried microparticles were also examined for their size distribution (see Figure S2, Supplementary Materials). The sizes of the PS21 microparticles spray-dried at an inlet temperature of 150 °C were in the range of 1–10 µm with a d_50_ value of 6 µm and a d_90_ value of 11 µm from the cumulative particle size distribution (Figure S2, Supplementary Materials). The results were consistent with sizes of particles observed using HIM. The microparticles were spheres or flattened spheres with a smooth, defect-free, non-porous surface (Figure 3b). After the milling process, the core of the microparticles was found to be hollow, indicating that the phages were encapsulated in the thin shell structure (Figure 3c). The red circle highlights the capsid heads of two phage virions protruding out of the shell matrix. Small “bumps” (indicated by the red arrow) on the surface of the shell may potentially be phage virions entrapped inside the shell structure (Figure 3d). These bumps were not visible in control particles not containing phages (Figure 3e). The thickness of the microparticle shell was estimated to be between 200 and 300 nm, which is similar in magnitude to phage dimensions (length ~200 nm). The images showed no merging of microparticles or any surface perforations (absence of blow holes).

### 3.3. Direct Compression Tableting of Spray-Dried Phage

Spray-dried microparticles were tableted using the process of direct compression. The spray-dried powder had suitable processing characteristics, i.e., flowability and hardness to undergo direct compression to produce tablets. The tablets had the following dimensions: diameter of 1 cm, thickness of 0.4 cm, and average weight of 0.3 g (Figure S3, Supplementary Materials). The tablets used in the study were all visually identical (any deformed or chipped tablets were discarded).

**Figure 4:**
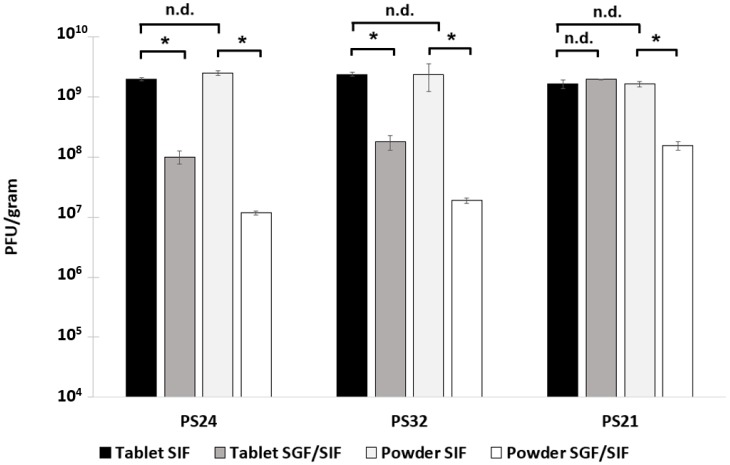
Encapsulated Felix O1 phages released in SIF from spray-dried powders and corresponding tablets using three different formulations. Samples labelled SIF were exposed to SIF only without acid exposure, whereas samples labelled SGF/SIF were exposed first to simulated gastric fluid (pH 2) for 2 h and were then centrifuged, before the supernatant was withdrawn and SIF was added to the sample. Phage titres were measured after 3 h of exposure to SIF for powders and 5 h of exposure to SIF for tablets to ensure complete dissolution of tablets. * Indicates significant difference in means (*p* < 0.05) using a two-sample *t*-test; n.d. means no difference (*p* > 0.05). Error bars represent one standard deviation; all measurements were done in triplicate (*n* = 3).

Powders containing different proportions of trehalose were evaluated in terms of acid stability following formation of tablets. The results showed there to be a significant difference between the three formulations (Figure 4). Post-spray-drying release in SIF showed titres of more than 1 × 10^9^ PFU/g in the spray-dried powders for all three formulations, which was similar in magnitude to the maximum theoretical phage titre yield (~1.7 × 10^9^ PFU/g) based on the phages present in the original spray-drying solution surviving the spray-drying process. The phage dose loaded in each tablet was approximately 6 × 10^8^ PFU per tablet, and no adverse effect of the compression force on subsequent phage viability was observed (Figure 4). A significant loss of phage titre was observed for all formulations in powder form after exposure to SGF (pH 2) for 2 h. For PS21, phage titre fell from 1.7 × 10^9^ PFU/g to 1.6 × 10^8^ PFU/g, i.e., ~1 log, whereas, for PS24 and PS32, the phage titre fell from ~10^9^ PFU/g to ~10^7^ PFU/g, i.e., ~2 log. The effect of tableting on acid protection resulted in marked improvement in acid stability for all three formulations. PS21 showed no observable loss in phage titre following 2 h of exposure to SGF (Figure 4). PS24 and PS32 showed only a ~1 log reduction compared with samples not exposed to SGF, along with a viable phage concentration in the tablets of 1 × 10^8^ PFU/g; this was ~1 log greater than that compared with PS24 and PS32 powders following exposure to SGF (~10^7^ PFU/g).

### 3.4. Storage Stability of Spray-Dried Phages in Powders

**Figure 5:**
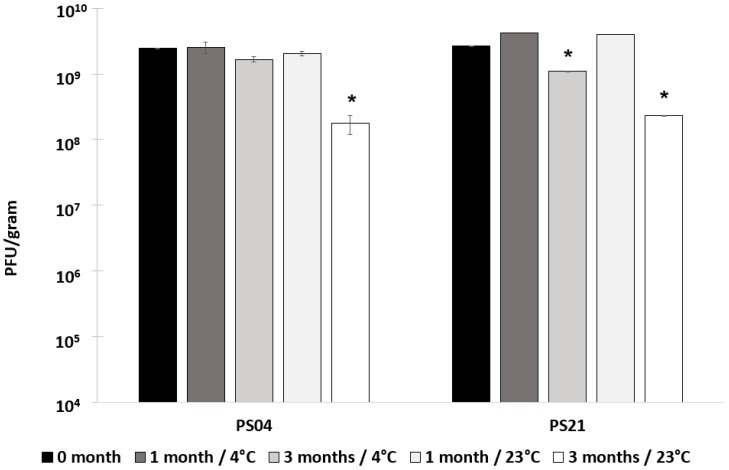
Storage results for formulations PS04 and PS21 used to spray-dry phage Felix O1. Phage titre was measured after one month and three months of storage at 4 °C and 23 °C. * Indicates a significant difference in means in comparison with the mean value immediately after spray-drying (0 months) using a two-sample *t*-test, (*p* < 0.05). Error bars represent one standard deviation; all measurements were done in triplicate (*n* = 3).

The most promising formulation in terms of acid stability (PS21) was compared with PS04 in terms of storage stability. Spray-dried powders had different initial moisture contents with PS04 3% (*w*/*w*) and PS21 9% (*w*/*w*) (Figure S1, Supplementary Materials). The powders were stored at two different temperatures (4 °C and 23 °C) for a period of three months. A significant loss in phage titre was observed after three months of storage for both PS04 (titre falling from 2.4 × 10^9^ PFU/g to 1.8 × 10^8^ PFU/g) and PS21 (titre falling from 2.7 × 10^9^ PFU/g to 2.4 × 10^8^ PFU/g) at 23 °C in comparison with the phage titre immediately after spray-drying (Figure 5). There was no statistical difference in phage titres for samples stored for one month at 23 °C for both PS04 and PS21 compared with titres immediately after production (0 months). There was no statistical difference in sample means for Felix O1 formulated in PS04 and stored at 4 °C for a period of three months (starting titre 2.4 × 10^9^ PFU/g, and 1.9 × 10^9^ PFU/g after three months). A slight decrease in phage titre was recorded after three months of storage at 4 °C for PS21 (titre falling from 2.7 × 10^9^ PFU/g to 1.1 × 10^9^ PFU/g).

## 4. Discussion

Trehalose was previously shown to protect phages during the spray-drying process [24]. The role of trehalose is both in stabilising phage protein conformation through hydrogen bonding and vitrification due to a high glass transition temperature of the spray-dried powder [29]. High residual moisture in samples spray-dried using relatively low drying temperatures results in lower glass transition temperatures and recrystallisation of the trehalose, which negatively affects phage stability [24]. Spray-drying Felix O1 phage formulated in pure trehalose resulted in no significant loss in phage titre at all spray-drying temperatures tested in the present study. The glass transition temperature of trehalose powders was previously correlated with residual moisture in the sample and amorphous trehalose samples, with moisture content typically below 5% (*w*/*w*) having T_g_ values greater than 50 °C [31]. The glass transition temperatures for PS04 samples were typically above 60 °C immediately following drying, due to low residual moisture content and the amorphous nature of spray-dried trehalose (Figure S4, Supplementary Materials). Phages encapsulated in a glassy matrix in samples having a low moisture content may result in better storage stability at low and ambient storage temperatures (Figure 5). It was not possible to reliably measure the glass transition temperatures of the composite trehalose–polymer PS21 microparticles. However, the moisture content of the samples was found to be dependent on the spray drying temperatures (Figure S1, Supplementary Materials). Spray-drying at a drying temperature of 150 °C resulted in PS21 powder having moisture content less than 10% (*w*/*w*) and high titres of viable phages in the dry powders. During the early stages of drying where the droplet surface remains saturated with moisture (100% relative humidity, RH), the droplet surface temperature is maintained at the wet bulb temperature, which is significantly lower than the hot air temperature. As drying progresses, the droplet temperature begins to increase as water diffusion to the droplet surface is not able to maintain 100% RH. As the air flow was co-current, the air temperature dropped due to evaporative cooling. Once the moisture content of the particles drops, the temperature of the particles begins to increase. However, the exposure period to high temperatures is fairly short due to the short residence times of the particles in the dryer. Felix O1 phages spray-dried in formulations containing ES100 and trehalose, e.g., PS21, remained viable at outlet drying temperatures as high as 82 °C. These results are particularly encouraging since industrial spray-dryers operate at similar temperatures (80–100 °C), which are markedly higher than those previously employed in published studies using small laboratory-scale dryers [25,32]. However, individual phages may show considerable differences in thermal stability and, therefore, spray-drying conditions may need to be optimised appropriately [32].

Spray-drying Felix O1 in a formulation containing only Eudragit S100^®^ (PS30) resulted in a significant loss in phage activity in the final dried powders (Figure 1) compared with spray-drying under identical conditions using pure trehalose (PS04). High residual moisture content in the polymer only powders coupled with the thermal stress may play an important role with respect to phage viability during spray-drying. In the present study, encapsulation of Felix O1 phage in a composite matrix containing different proportions of trehalose and Eudragit S100^®^ resulted in pH-responsive microparticles with good retention of high titres of viable phages (~10^9^ PFU/g) in the spray-dried powders. Addition of trehalose in the polymer formulation afforded phages significant protection from the thermal and desiccation stresses encountered during spray-drying compared with phages formulated in the polymer without any trehalose present. PS21 powders had lower moisture content compared with PS30 (Figure S1, Supplementary Materials). A higher proportion of trehalose in the formulation resulted in higher phage titres, e.g., PS32 (40% (*w*/*w*) trehalose) was higher than PS21 (33% (*w*/*w*) trehalose). The particle morphology of PS21 powders was spherical and defect-free (Figure 3). The particles were hollow internally and had an outer skin with phages presumably encapsulated within the shell (Figure 3). The absence of blow holes suggested that the drying temperature and rate of drying were not excessive and, consequently, did not result in an increase in the internal water pressure to burst the microparticles. Formulations resulting in a high proportion of trehalose in the structure of the composite microparticles (PS32 and PS24) showed poor acid stability (Figure 2). However, increasing the proportion of polymer in the microparticle shell (PS21) enhanced acid protection for the phages exposed to SGF at pH 2 for 3 h (Figure 2 and Figure 4). Acid stability of the spray-dried phage powders was improved considerably by forming tablets using a direct compression tabletting process routinely used in the pharmaceutical industry (Figure 4). The physical properties such as bulk density, brittle fracture, and plastic behaviour of the spray-dried microparticles were suitable for the formation of robust tablets (Figure S3, Supplementary Materials). The process of direct compression did not adversely affect the phage titre (Figure 4). The proportion of trehalose in the formulation affected phage acid stability in the tablets with the high polymer-containing formulation (PS21) showing complete acid protection (Figure 4). Felix O1 phages were stable in formulation PS21 stored at 4 °C over a period of three months. The moisture content of the PS21 powders was higher ~9% (*w*/*w*) compared with trehalose-only powders (PS04) under similar drying conditions, which may have adversely impacted on storage stability (Figure S1, Supplementary Materials). Future work will evaluate phage stability in PS21 powders dried at 180 °C which had low moisture content similar in magnitude to PS04 (Figure S1, Supplementary Materials). Optimisation of the proportion of trehalose and Eudragit S100^®^ was needed to ensure good thermal protection for the phages, attributed to trehalose during the spray-drying process, whilst ensuring acid protection due to the presence of high amounts of polymer in the microparticle shell encapsulating the phage. Formulation PS21 was found to be superior to PS24 and PS32 in terms of protecting phages from acid exposure after forming tablets using direct compression. Formulation PS21 afforded phage protection during thermal spray-drying, resulting in dry powders with high viable phage titres showing good storage stability. PS21, therefore, fulfils the criteria of a suitable formulation for production of acid stable oral solid dosage forms using spray-drying.

Spray-drying is a highly scalable industrial process which is suitable for manufacturing encapsulated phages in aqueous polymer formulations, such as the one evaluated in the present study. The spray-dried powders were spherical with good mechanical and flowability properties and could be reliably tabletted into oral solid dosage forms suitable for enteric delivery. One limitation of the present study is the short residence times and small particle sizes achieved using small laboratory-scale spray-dryers. Bench-top spray-dryers are only capable of producing small particles, typically <10 µm, similar to those produced in this study, which require drying times of only a few seconds. Industrial-scale dryers produce larger particles ~50 µm and, therefore, have considerably longer residence times [28]. Future work needs to evaluate the stability of the phages produced using a pilot-scale spray-dyer resulting in larger particles.

The tableted phages would allow ease of use (good for patient compliance) and reliable delivery of high titres of viable phages at the site of infection in the gastrointestinal tract. These are important advantages and can be achieved using the relatively simple, highly scalable, and low-cost process evaluated in the present study. Phage-containing tablets in standard blister packs would need to be stored under refrigerated conditions, which is not ideal; therefore, further work is needed to improve the formulation such that the tablets can be stored without the need for a cold supply chain. Future work is also needed to evaluate the in vivo release characteristics of the phage-containing tablets and the targeted delivery of phages at specific locations in the gastrointestinal tract.

## Supplementary Materials

The following are available at https://www.mdpi.com/1424-8247/12/1/43/s1.
