# Supplementary material for: Microencapsulation of Salmonella-Specific Bacteriophage Felix O1 Using Spray-Drying in a pH-Responsive Formulation and Direct Compression Tableting of Powders into a Solid Oral Dosage Form
Source: Pharmaceuticals (Basel). 2019 Mar 22;12(1):43. doi: 10.3390/ph12010043 (PMC6469172; doi:10.3390/ph12010043)
Supplement: Supplementary file 1 [file pharmaceuticals-12-00043-s001.pdf]

Supplementary Information

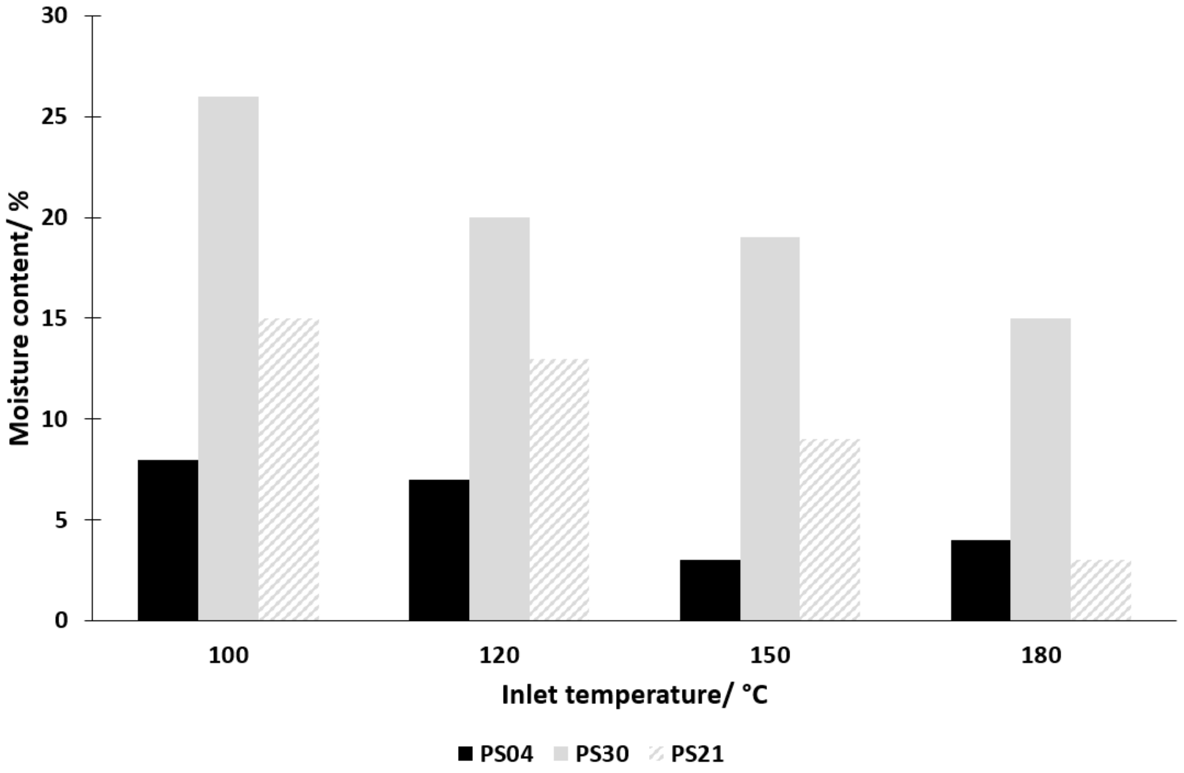

Figure S1. Moisture content of spray dried powders at different drying temperatures. All measurements were done in triplicate.

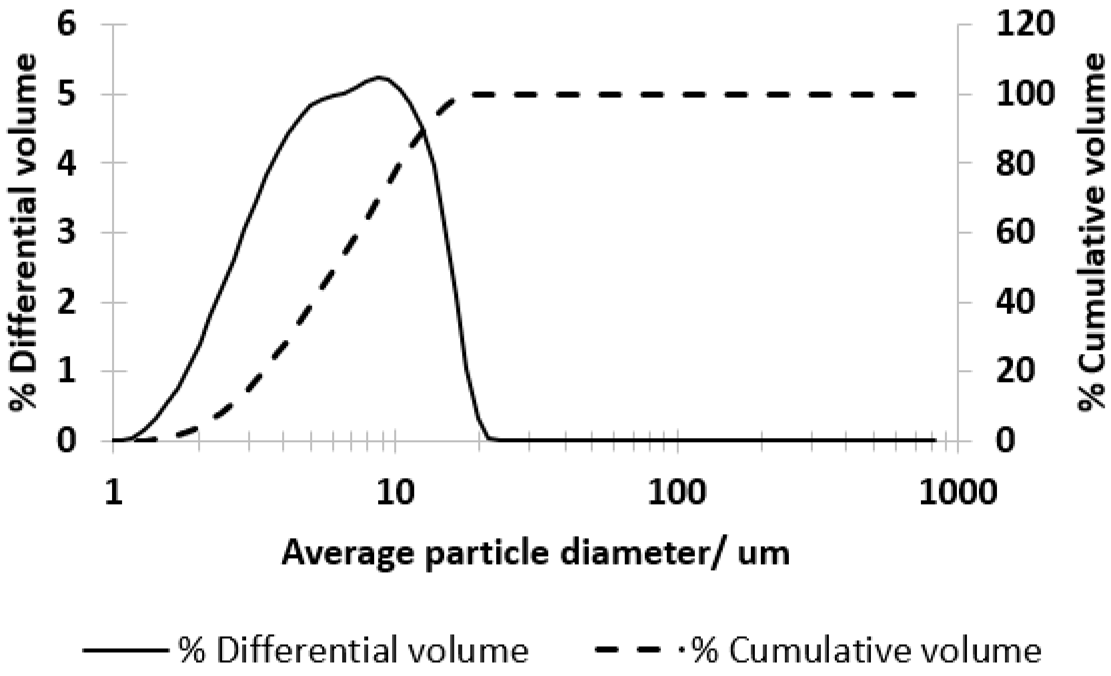

Figure S2. Coulter particle size distribution measurements for PS21 spray dried powder using inlet temperature 150°C. Differential volume distribution of particles (solid line), cumulative volume distribution (dashed line). Measurements were done in triplicate.

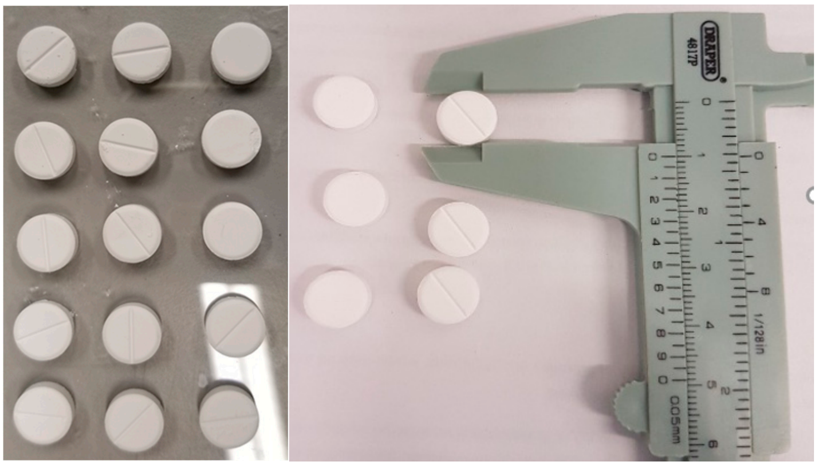

Figure S3. Images of tablets produced using the process of direct compression on spray dried powders PS21.

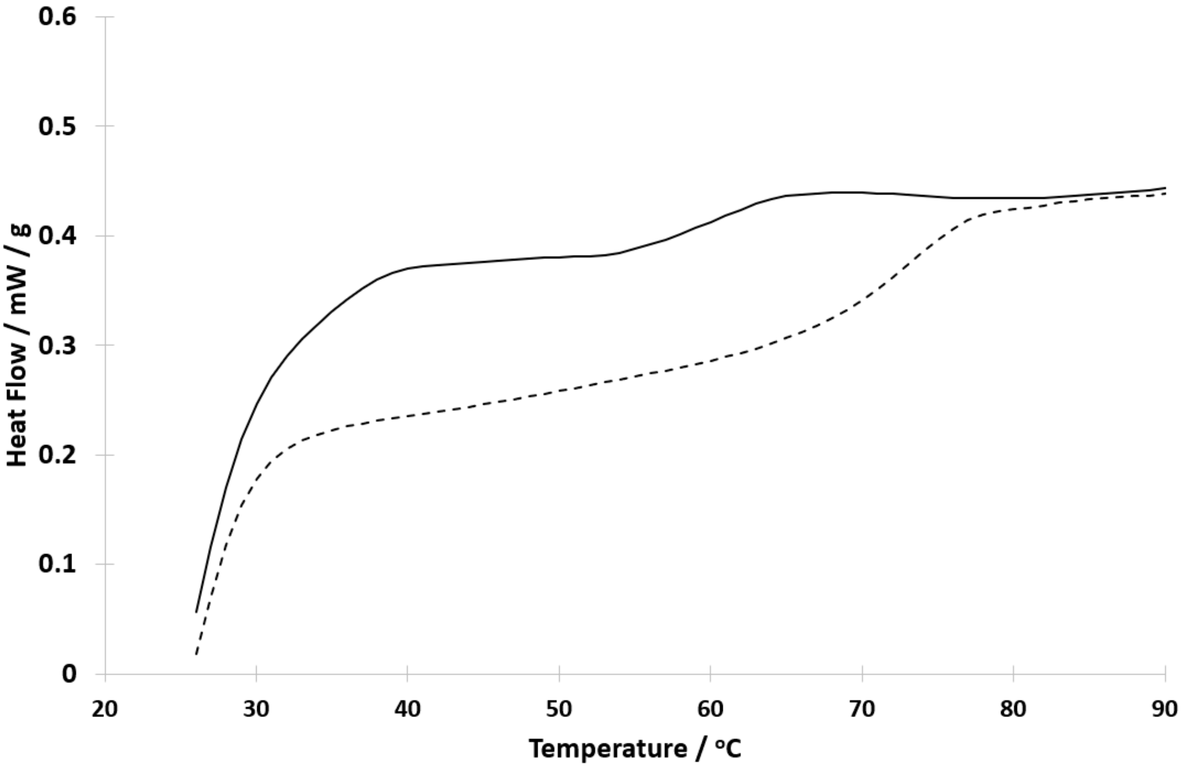

Figure S4. Differential Scanning Calorimetry of spray dried trehalose powders spray dried trehalose powders (PS04) showing the effect of spray drying inlet temperature 120 ^{o}C (solid line) and 150 ^{o}C (dashed line), on glass transition temperature.
